# Enhancing contact recommendation in social platforms through mental health awareness: Exploring Anorexia Nervosa as a case study

**DOI:** 10.1371/journal.pone.0312766

**Published:** 2025-02-10

**Authors:** Diana Ramírez-Cifuentes, Ricardo Baeza-Yates, Meritxell Lozano, Ana Freire

**Affiliations:** 1 Department of Information and Communication Technologies, Universitat Pompeu Fabra, Barcelona, Spain; 2 Computer Vision Center (CVC), Bellaterra (Cerdanyola del Vallès), Barcelona, Spain; 3 Institute for Experiential AI, Northeastern University, Boston, MA, United States of America; 4 Fundación Instituto de Trastornos Alimentarios, Barcelona, Spain; 5 UPF Barcelona School of Management, Barcelona, Spain; Ben-Gurion University of the Negev, ISRAEL

## Abstract

We analyze and propose a solution for the exposure of vulnerable users to harmful content during their interaction with contact recommender systems in social platforms. Our approach is dedicated to maximizing the number of harmless accounts suggested to users at risk. For these users, the over-personalization of recommender systems can result in an exposure to triggering content. We consider anorexia nervosa as a use case. People with anorexia tend to seek accounts of peers that support their unhealthy habits. Contact recommender systems can unintentionally reinforce such behaviors. Our approach modifies the objective function of a content and topology-based recommendation algorithm to maximize the suggestion of harmless accounts for users at risk. This is done with data from Twitter of Spanish speaking users with anorexia. The design and evaluation of the proposal has involved the participation of clinicians and volunteers at the last stages of treatment. Results show that users with anorexia are willing to follow harmless accounts suggested in online platforms. There is a tradeoff in precision (Pr) when comparing our proposal (Pr = 0.41) with a regular recommendation approach (Pr = 0.58). However, results are promising as there is a 55% increase in the percentage of harmless accounts suggested.

## 1. Introduction

Mental health challenges, particularly eating disorders like Anorexia Nervosa (AN), significantly impact eating behaviors and self-perception, often leading to severe weight loss [1]. People with eating disorders often turn to social platforms for weight loss tips and peer support, reinforcing unhealthy behaviors [2, 3]. Researchers utilize machine learning to analyze these users’ social media activity for screening tasks—evaluating digital content to detect early signs of conditions like eating disorders [3–9].

Recommender systems have grown significantly over the last few years, resulting in advanced and specialized approaches, which vary according to purpose, domain, and degree of personalization [10]. In this paper we address contact recommender systems, which are designed to suggest social connections or contacts to users within a digital platform, aiming to enhance networking and social interaction by identifying potentially valuable connections based on shared interests, mutual connections, or behavioral patterns [11].

Recommender systems on social networks can lead to over-personalization, narrowing our exposure to diverse information and isolating us from different societal groups [12]. This is particularly risky for individuals with mental health issues like depression or eating disorders, as they might be directed to harmful content [13], exacerbating their conditions [14–16].

In social platforms, prior studies have identified two types of communities related to eating disorders: ED communities and pro-recovery communities. They have found that among these communities the communication is mostly intra cluster [17]. However, a shift in the interests of users as they move towards treatment has been found, suggesting that the exposure to pro-recovery content might not lead to its rejection [18].

**Fig 1:**
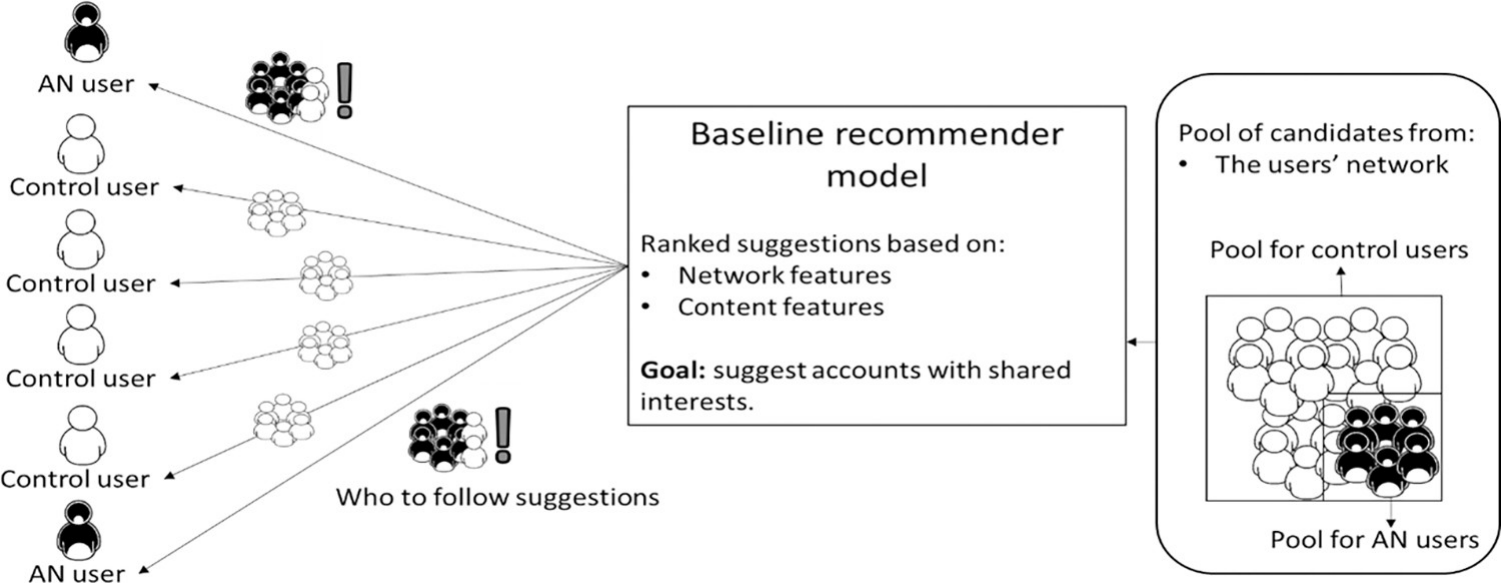
Architecture of common contact recommendation models. Referred here as a Baseline recommender model, which is potentially harmful for vulnerable users.

We propose a contact recommendation approach suitable for social platforms where users can establish links with others through a follow relation. Twitter is an instance of such platforms where, given a user *u*, the users followed by *u* are referred as *u’*s *followees*, whereas the users following *u* are referred as *u*’s *followees*. As it can be seen in Fig 1, the objective of a common recommendation model [19] is to rank on top the accounts that the user is more likely to follow, under the principle that people tend to follow users who they are likely to know (network) or that have interests in common (content). As users with AN are more likely to be following their peers or accounts that promote unhealthy habits (harmful accounts) [17], it is likely for the recommender to provide harmful suggestions as we will later prove.

Social platforms can support recovery by offering pro-recovery communities that provide emotional support [20], yet they also risk becoming echo chambers that isolate users from dissenting views, particularly in ED communities [21]. Our work introduces a social recommender system for AN, designed to avoid harmful content and promote beneficial connections, thereby enhancing inter-cluster communication without being limited to pro-recovery suggestions alone. We aim to mitigate the risks of echo chambers and filter bubbles [22] by recommending safe and relevant content to users.

In prior work [18, 23], the recovery process from AN was mapped to the trans theoretical model of health behavior change (TTM). It describes the progress of people toward adopting and maintaining healthy behaviors. The model consists of 6 stages of change, including the contemplation stage, where people are conscious of an existing issue, yet they simultaneously consider and reject changing their unhealthy habits. This stage is relevant to define our recommendation approach, as users at this stage are more likely to look for help, which might eventually lead them to reach out for proper treatment.

With the context and relevance of the proposal described, we formalize our research questions as follows: RQ1) which are the main terms and topics of interest addressed by people with Anorexia Nervosa at the contemplation stage? And how do the interests extracted from social media data differ from those provided by volunteers through surveys? RQ2) Can users at the contemplation stage be automatically detected in a social platform context? And how? RQ3) Can user accounts be automatically labeled as harmless in a social platform context? And how? RQ4) What is the percentage of harmful and harmless accounts suggested by Twitter’s recommender system to AN users? RQ5) Can a contact recommendation approach minimize the ratio of harmful accounts suggested to a user with AN? RQ6) How likely are target users to follow the accounts recommended by our approach, and how effective is such approach compared to common recommendation methods? RQ7) How to evaluate that a model maximizes the number of accounts followed and also gives relevance to the selection of non-harmful accounts?

With the prior questions in mind, our main contributions are the following: 1) an analysis of the interests of people with AN at the contemplation stage within social platforms; 2) the definition of a harmless contact recommendation approach for users with AN; 3) a classification model to detect users at the contemplation stage; 4) a classification model to distinguish harmful from harmless accounts; 5) an evaluation approach that involves the participation of experts, and volunteers with AN; 6) the definition of a measure that evaluates the performance of the recommendation approach taking into account its precision and the ratio of harmless accounts followed.

## 2. Methods

### 2.1. Data description and ethical concerns

The dataset employed in this study was meticulously curated to ensure the integrity of the research while upholding the highest ethical standards. The data consists of anonymized and aggregated information extracted from a collection of posts (multiple posts per user) of random (Spanish speaking) Twitter users, and users identified to be at the contemplation stage of Anorexia Nervosa (AN) [18].

The collection and analysis of the data were conducted in strict adherence to the guidelines set forth by the Institutional Committee for Ethical Review of Projects (CIREP) at Universitat Pompeu Fabra, with approval number 162. This process involved the application of a harmless users’ detection model and the inclusion of pre-labeled pro-recovery accounts to create a pool of candidates for our recommender system architecture. A considerable emphasis was placed on minimizing the risk of harm by prioritizing the suggestion of content that supports recovery and well-being.

To ensure the compliance of our data collection and analysis methods with the terms and conditions of Twitter, we followed a rigorous protocol that involved only accessing publicly available data, using Twitter’s API in accordance with its usage policies. Our methodology, described in detail in Section 2.2, reflects our commitment to ethical research practices and the protection of individuals’ privacy​. For evaluation purposes, surveys were conducted over voluntary participants that have recovered from Anorexia Nervosa. They provided written consent before filling the surveys.

Our findings are relevant for the development of recommender systems that do not expose users to harmful content. However, before releasing these tools, it is crucial to assess their risks and benefits and ensure compliance with legal standards to prevent misuse. Following Institutional Review Board (IRB) guidelines, this research strictly upholds confidentiality and privacy protections, limiting data sharing to ensure participant information security and uphold the study’s ethical standards. Consequently, access to this project’s data is restricted.

### 2.2. Recommender system architecture

**Fig 2:**
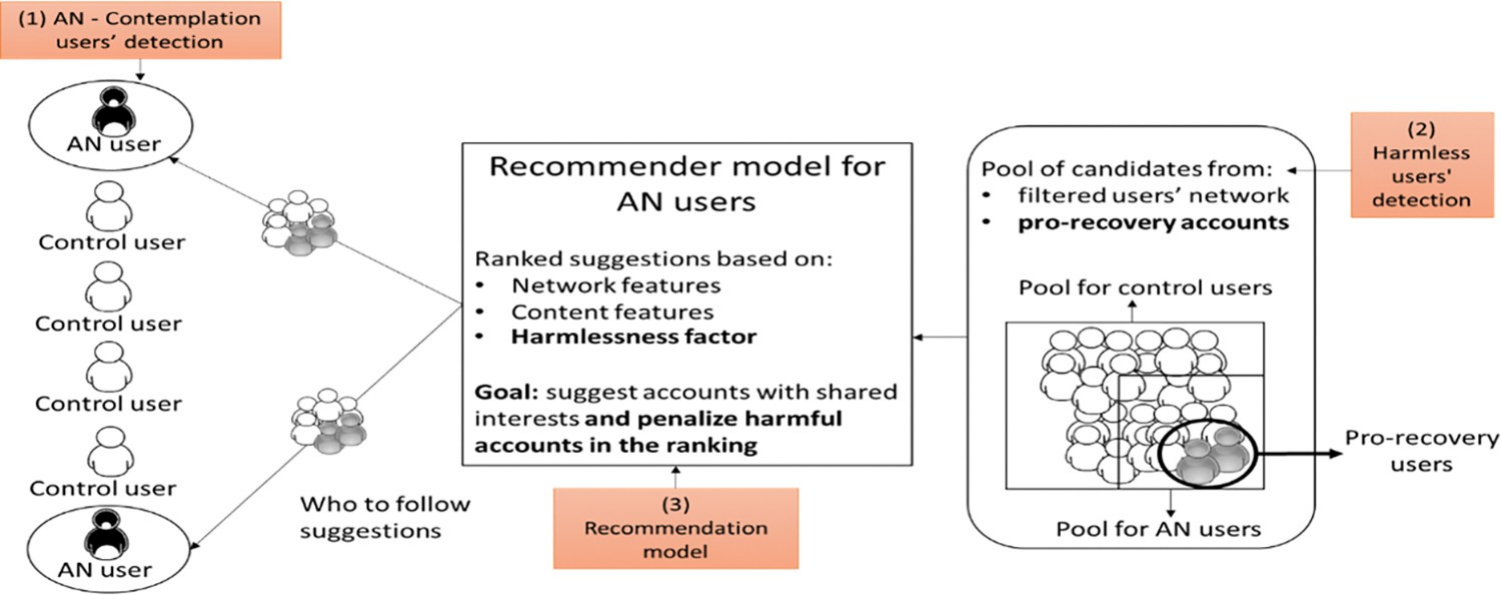
Architecture of the recommender system proposed.

Our recommendation approach (RQ5) (Fig 2) consists in 1) detecting AN users at the contemplation stage as the recommendation approach will be applied exclusively over such users (Section 2.3); 2) defining a pool of candidates composed by users that are more likely to be harmless. This is done by applying a harmless users’ detection model (Section 2.4) for the definition of the pool of candidates and by introducing a group of pre-labeled pro-recovery users to the pool. Finally, 3) the recommendation model’s objective function (Section 2.5) is defined by a combination of network and content scores with a weight given by a harmlessness factor, which modifies the score of the suggested candidates by penalizing those that are likely to be harmful. Users are ranked according to the score obtained, and the top *K* suggestions are displayed to the user. This approach also makes sure that some pro-recovery accounts are part of the suggestions displayed. Depending on the top *K* suggestions that will be showed to the user, a fixed percentage of these should correspond to those pro-recovery users with the highest scores obtained (based only on the content score).

**Fig 3:**
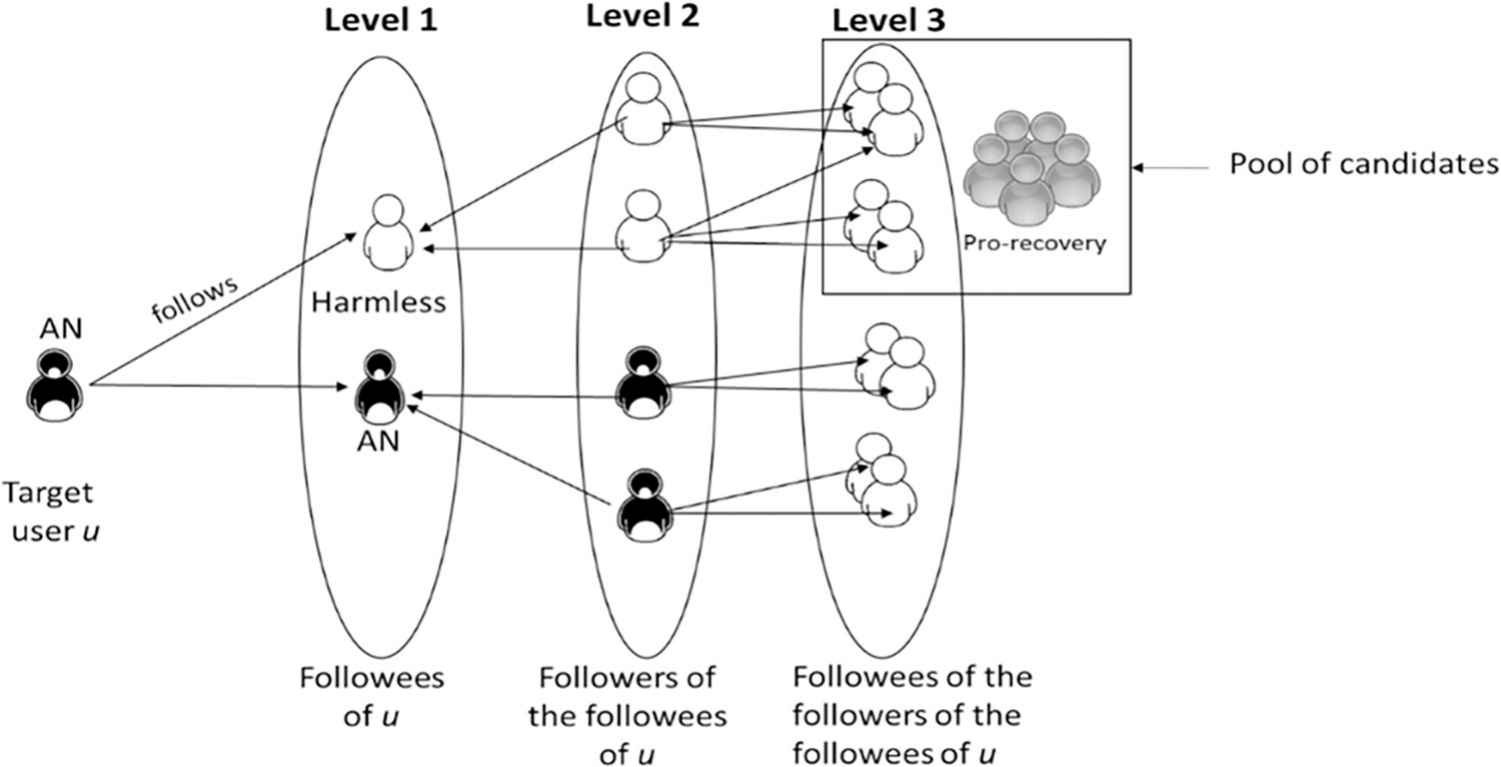
Definition of the pool of candidates for an AN target user *u*.

The pool of candidates of a target user *u* is given by their neighborhood as described in [24], meaning that, according to Fig 3, it is defined by users from level 3. Considering that through this way most of the users in the pool would be harmful, we do a prior filtering step, where we apply a classifier to detect harmless users over u’s followees (Level 1 users) to reduce the likelihood of suggesting harmful accounts.

Notice that through this study, asides from contemplation and control accounts we address 1) harmful accounts, which are those that can negatively influence the behavior of users with anorexia, here we can find accounts that promote diets and excessive exercising, accounts that express concerns about body image and promote unhealthy eating habits, and especially pro-ED accounts, among others. 2) Pro-recovery accounts that correspond to specialized recovery centers, educational psychologists, foundations, and people that can offer support and information towards recovery from eating disorders. 3) Neutral accounts that do not promote harmful nor pro-recovery content. Finally, we consider 4) harmless accounts, which correspond to the union of neutral and pro-recovery accounts.

### 2.3. Contemplation users’ detection

For the recommendation approach to target only users at the contemplation stage of AN (RQ2), we developed a machine learning-based predictive model which was trained using features extracted from a sample of users at the Contemplation stage of AN. To create this model, 2 clinicians were asked to label a set of 171 (user-level) Twitter writings of people with AN that were going through the precontemplation or contemplation stages according to the TTM.

The dataset used for this purpose is described in [18]. This dataset comprises Spanish tweets from a year-long period ending December 21, 2018, focusing on eating disorders. It was curated using keywords and hashtags from various sources, including pro-anorexia blogs and academic studies. Users were categorized by mental health professionals into groups such as those experiencing anorexia (AN group), which include precontemplation and contemplation stages; users undergoing treatment; recovered users; and control groups, ensuring a comprehensive representation of the disorder’s spectrum according to the trans theoretical model of health behavior change [18].

Within the AN group, the precontemplation stage is when individuals may not acknowledge their behavioral issues, making them less receptive to assistance. Thus, our focus is on those in the contemplation stage, who are beginning to recognize their problems and are more open to seeking help. We also exclude individuals already in treatment, assuming they are actively pursuing safer content under clinical guidance.

After the labeling process, clinicians defined as contemplation cases the ones for which both annotators agreed (56 cases). Thus, contemplation was assigned as the main target class to predict versus a group of newly collected random control cases (498 cases). The remaining AN users at the precontemplation, treatment and recovered stages were not taken into account for the model.

For the detection of contemplation cases, we evaluated multiple predictive models with several features including a 1) TF-IDF (Term Frequency-Inverse Document Frequency) bag of words model (BoW), where user representation is based on the occurrence frequency of 1–3 grams (all included) in their writings adjusted by the inverse frequency across all users to highlight unique terms [25], the model used a total of 94,488 features considering that a user was represented by the concatenation of the texts of its publications collected. 2) Corresponds to a features’ model named lexicon model where we extract attributes from the texts trying to map the characteristics that are often observed by clinicians for AN screening. The features were mainly gathered from the content shared and interests of the users. These features consist in 84 features defined by linguistic and psychological aspects through the following categories: linguistic dimensions (24 features); affective processes and emotions (29 features); personal concerns and biological processes (12 features), vocabulary related to suicide risk factors (10 features) and vocabulary related to eating disorders (9 features). These features are described in [18]. Each of these models (TF-IDF and lexicon-based) were used for the task using multiple classification methods such as Logistic regression (LR), random forest (RF), and Support vector machines (SVM), with 5-fold cross validation and applying SMOTE’s [26] oversampling method to overcome imbalanced data issues. 3) The third type of model uses word embeddings and feeds them as an input for a deep learning approach that uses Convolutional Neural Networks (CNN) based on the approach described in [27]. This model uses for the input word embeddings provided by [28], they were learned over a dataset with 2 million Spanish tweets. As described in [28], the embeddings were initially generated through pre-training on a multi-lingual dataset that did not require direct supervision for each language. These pre-trained embeddings were then fine-tuned during the training of the specific model to adapt them to the particular task of sentiment classification. Later, these word embeddings were finetuned on our AN the training set. For the CNN, the embeddings were fed as input, which involves converting the text into sequences of tokens using a tokenizer, and then transforming these sequences into dense vectors using the embeddings. The resulting matrix, composed of these vectors, represents each word in the text as part of the input to the CNN. This matrix is then fed into the CNN, where convolutional layers process the embedded word representations. A filter window covering 2, 3, or 5 terms was employed, followed by max pooling. The resulting output was directed to a Sigmoid layer, producing the final predictions. The method was evaluated averaging the results of several runs, with a validation set of 10% of the training samples (70% of all the instances) in each run.

All the models were evaluated in a test set which corresponds to the remaining 30% of all the data instances. We evaluated the performance of the models proposed in terms of Precision, Recall, and F1-Score for the main class to predict, and Accuracy for both classes.

Our inclusion of a CNN model was intended to explore a range of methodologies, including those prevalent in current research trends. However, it is important to note that within our study, the predictive models were designed with a focus on explainability, prioritizing features that clinicians can readily interpret (vocabulary used and lexicons) [29]. Given that our primary aim is not to perfect predictive modeling but to demonstrate a feasible architecture for identifying contemplation cases in eating disorders, the models presented should be viewed as reference points. They fulfill a crucial role in our system’s architecture, aligning with our objective to provide actionable insights while maintaining the transparency and interpretability essential for clinical applications.

### 2.4. Harmless users’ detection

Since our approach filters user’s followees to consider those that are more likely to be harmless for the pool of candidates, we created a classifier capable of distinguishing harmful from harmless accounts (RQ3). For this purpose, we used the dataset described in [18]. We labeled control accounts that included pro-recovery accounts among them, as either harmless, harmful, or doubtful (for those cases where annotators were not sure about their choice). We also assigned to the cases labeled as AN of the dataset the ‘harmful’ label. These cases are the contemplation and precontemplation ones corresponding to the 171 AN cases in the dataset [18]. They are considered as harmful because our intention is to not recommend contacts likely to be sharing pro-AN content.

We developed a harmful vs. harmless cases classifier. We adopted the same approaches described for the contemplation users’ detection to create our predictive models. The main target class assigned was the harmless one. The same evaluation approach and measures as for the contemplation users’ classifier were used. This classifier is relevant as also it is used to calculate what we later define as the harmlessness factor (Section 2.5.3).

### 2.5. Candidates ranking algorithm

Among the pool of candidates for a given target user *u*, we rank candidates based in a comparison between *u* and each of the candidates *c*_*x*_ to be recommended. We use similarity measures to suggest candidates that are more alike, in terms of shared interests (content), and the user’s network topology [19, 24, 30]. In addition to these common elements, we propose a harmlessness factor, which ranks recommendations based on how harmless for the user the candidate is likely to be. The elements considered to obtain a ranking score for each candidate, given a pair (target user *u*, candidate *c*_*x*_), are defined by the following elements:

#### 2.5.1. Topology attributes

We consider two elements: 1) as it is likely for users of level 2 (see Fig 3) to have followees in common, we measure the number of times the candidate *c*_*x*_ appears in the pool of candidates *C*_*u*_ of the user over the total number of existing candidates in *C*_*u*_ (Eq 1). Notice that for our experiments we defined each pool to have 100 random candidates among the eligible users. The next element is given by 2) the followees in common between *c*_*x*_ and *u*, which is defined by the calculation of Jaccard’s similarity [25] between the set of followees of *u* and *c*_*x*_ (Eq 2). A similar method is used in [31] but they only consider the size of the intersection between the sets of followees of *u* and *c*_*x*_. Finally, a topology score (Eq 3) is given by the average of both scores.


$$Ocurrences\_ratio(u,c_{x})=\frac{{\#(c}_{x},C_{u})}{\left|C_{u}\right|}$$ (1)

$$Jsim(u,c_{x})=\frac{\left|Followees(u)\cap Followees(c_{x})\right|}{\left|Followees(u)\cup Followees(c_{x})\right|}$$ (2)

$$Topology\_score(u,c_{x})=Avg(Jsim(u,c_{x}),Ocurrences\_ratio(u,c_{x}))$$ (3)

#### 2.5.2. Content attributes

We compare the interests of each candidate *c*_*x*_ with those of the target user *u*. Our goal is to recommend candidates that have more shared interests with *u*. To define a measure of the interests of a given user, we followed the approach described in [18] considering that the topics of interest of a user are given by (1) the content posted by themselves, (2) the content they like (given by the tweets made by others and marked as favorites) and (3) the interests of their followees. For each user, we collected (1) a random sample of their own tweets (up to 200 texts), (2) a random sample of 200 tweets that they had liked during the same period, and (3) the profile descriptions (biographies) of up to 200 random followees of the user. The resource used to extract the topics was Dandelion’s entity extraction API [32], which given a text, it extracts key n-grams and returns Wikipedia’s and DBLP’s categories to which a term or n-gram belongs to, providing semantic categories.

In our approach, we first mapped the topics of interest for all users into a bag of words model, creating a unique vector for each user that quantifies their interest in various topics. The relevance of each topic to a user was normalized on a scale from 0 to 1, based on the highest (max) and lowest (min) topic scores observed across all users. This normalization ensures that each user’s topic interest vector *v*_*u*_​, reflects their relative interest intensity in the context of the broader user base.

To assess the similarity between a target user *u* and a candidate *c*_*x*_, we compared their respective interest vectors, *v*_*u*_ and $v_{c_{x}}$, using cosine similarity [24]. This method calculates the cosine of the angle between the two vectors, providing a measure of how closely the topics of interest for *u* align with those of *c*_*x*_. The formula for the content score between user *u* and candidate *c*_*x*_ is given by Eq 4:

$$Content\_score(u,c_{x})=CosSim(v_{u},v_{c_{x}})=\frac{v_{u}.v_{c_{x}}}{{\|v}_{u}\|\times \|v_{c_{x}}\|}$$ (4)

For instance, consider two users, *u* and *c*_*x*_, with interest vectors based on topics like “nutrition,” “fitness,” and “well-being.” Suppose *u* ‘s interest vector is *v*_*u*_ = [0.8,0.1,0.5] and *c*_*x*_’s vector is $v_{c_{x}}$ = [0.7,0.2,0.4], where each value represents the normalized interest level in the respective topics. The content score​ is calculated using Eq 4. In this example, the calculation would reveal a score that quantifies the degree of similarity in their topic interests, suggesting *c*_*x*_ as a potentially relevant connection for *u* based on shared interests.

#### 2.5.3. Harmlessness factor

We introduce a harmlessness factor, which penalizes harmful accounts in case they are part of the pool of candidates. This factor is given by a harmlessness score, which is represented by the output (probability estimates) of the harmlessness classifier. The score is between [0,1] recalling that the higher the score, the less harmful the candidate is.

Finally, the rank score for *u* and *c*_*x*_ is given by Eq 5.


$$Rank\_score(u,c_{x})=harmlessness\_score(c_{x})\times Avg(Content\_score(u,c_{x}),Topology\_score(u,c_{x}))$$ (5)

Notice that for the pro-recovery candidates, the rank score is given only by the product between the harmlessness and content scores.

### 2.6. Evaluation methods

We evaluate the viability of our proposal with volunteers, further referred as *survey participants*, that have gone through the contemplation stage of AN. We also do an annotation-based evaluation of the proposal, considering users’ data (RQ6).

#### 2.6.1. Survey participants’ evaluation

From May 3, 2021, to January 31, 2022, we involved 22 AN participants from a recovery center, bypassing social media data. The process involved: 1) gathering their contemplation phase interests via surveys; 2) aligning these interests for comparison with potential Twitter recommendations; 3) using a modified rank score to suggest user connections (Eq 6); and 4) evaluating their willingness to follow the top 5, 10, and 15 recommendations. The candidate pool, detailed in Section 2.6.2, comprised 1,491 unique users from the Twitter user methodology.


$$Participants\_rank\_score(par,c_{x})=harmlessnes\_score(c_{x})\times Content\_score(par,c_{x})$$ (6)

To discern participants’ interests, we analyzed Twitter users at the contemplation stage (Section 2.5.2), extracting their top 200 topics of interest in general and categorizing them for a survey (Table 1). Participants rated each subcategory as low or high (0–5) based on their contemplation phase level of interest, adding specific examples like “video games,” along with specific interests (“Mario Kart”). They also assessed the connection of these interests to AN and evaluated the potential harm of platform recommendations.

**Table 1:** Categories and subcategories defined from the topics of interest of contemplation users.

Main categories	Subcategories
Technology	• Applications, social networks, and social media social media • Technological devices • Videogames
Health	• Nutrition • Physical wellbeing • Mental wellbeing
Lifestyle and personal beliefs	• Interpersonal relationships • Activism • Religion and spirituality • Economy • Politics and justice
Science	• Philosophy • Sociology • Biology • Cosmology • Chemistry • Physics and Mathematics
Hobbies and entertainment	• Sports • Movies and TV • Music • Literature
Other interests	• Current news • Languages • Cultures of the world • Others

We gathered participants’ interests using their provided keywords for designated categories. For example, if a participant rated their interest in the “video games” category as 4 and mentioned “PlayStation” as a specific interest, we applied this interest level of 4 not only to “PlayStation” but also to related concepts like “game console,” “video games,” and “Sony consoles.” This method allowed us to create a comprehensive interest profile for each participant, represented as a scored vector within a bag of words/topics model, with scores normalized between 0 and 1 based on the participant’s highest and lowest topic scores.

For Twitter candidates, we analyzed their profiles to compile a similar vector of topics based on the frequency of topic-related keywords. The normalization process was applied here as well, ensuring a consistent comparison framework. By aggregating all participant topics, we established a basis for comparing the topic vectors of participants and candidates, using the participants’ rank score to prioritize candidate recommendations effectively.

*a) Survey participants’ evaluation baselines*. In addition to our approach, we defined 5 baselines for recommending users with which we compare our recommendation approach. They are described in Table 2, where we define recommendation methods, types of users of the pool of candidates, and ways for obtaining the pools of candidates. Notice that neutral accounts correspond to users that are not harmless but do not share pro-recovery content either. We can see that the pool of candidates defined for model V.4 has several harmful candidates, while this changes when the filtering approach of our method is applied (model V.5). Our model differs from model V.5 given that in addition to the content score, we consider the harmlessness score, precisely with the intention to rank at the top those harmless users that share interests with the participants. Moreover, our method introduces beneficial accounts in the pool of candidates given that it is less likely for these types of accounts to make it to the pool.

**Table 2:** Baselines defined for the evaluation of the participants.

Baseline model	Source of pool candidates	Types of users considered for the pool	Rank score per candidate of a given participant (par)
Model V.1	Sample of twitter accounts that were labeled as harmful.	Only harmful users	*Content*_*score*(par,*c*_*x*_)
Model V.2	Sample of twitter accounts that were labeled as pro-recovery.	Only pro-recovery users	*Content*_*score par*
Model V.3	Sample of twitter accounts that were labeled as either harmful, neutral, or pro-recovery.	Equal number of pro-recovery, harmful and neutral users	Random suggestions
Model V.4	Sample of users obtained from the pool of candidates of the user’s evaluation approach without considering the filtering step of our method. This would be equivalent to a state-of-the-art method of obtaining pool candidates.	Pro-recovery (0%), harmful (82%) and neutral (18%) users.	*Content*_*score*(par,*c*_*x*_)
Model V.5	Sample of users obtained from the pool of candidates of the user’s evaluation approach considering the filtering step of our method.	Pro-recovery (0%), harmful (30%) and neutral (70%) users.	*Content*_*score*(par,*c*_*x*_)

*b) Evaluation measures*. To assess our model and the baselines, we presented participants with the top 15 candidate suggestions from each model, including 20% pro-recovery users. Participants indicated who they would follow during the contemplation phase. We measured precision (P)–the fraction of suggested users that participants would follow, recall–(R)- the fraction of actual follows among all potential follows, and mean average precision (MAP) at 5, 10, and 15 recommendations, considering the ranking accuracy for preferred users [25]. Notice that for recall and precision we report the average of the results of all the participants. Also, given that we evaluated several models with the participants, they were only asked to choose who to follow among the top 15 users recommended by each model. In addition to these common measures that evaluate the likelihood of a participant to follow a recommended user, we also measure the ratios of harmful, neutral, pro-recovery and harmless (neutral + pro-recovery) users recommended by each model (#accounts of a given type suggested at K / K); along with the ratio of users of each of these types that would actually be followed over the number of suggested users of each type (#accounts of a given type followed at K / #accounts of a given type suggested at K). We also evaluate the ratio of accounts of each type followed at K (#accounts of a given type followed at K / K).

Finally, considering that a good recommendation model should maximize the average precision (AP) [25], and the ratio of harmless accounts followed for a given target user, we define an evaluation measure that aggregates both scores (RQ7). The score denoted as the Average Precision-Harmlessness Ratio Score (APHR) for a target user is given by the harmonic mean between the average precision and the ratio of harmless users followed at K (# harmless users followed at K/K) denoted as HLFRK, as it can be seen in Eq 7. We consider the harmonic mean to be adequate as it would strongly penalize the cases where only harmful accounts are suggested. Also, to calculate this measure for all target participants o users, the MAP and the average of the HLFRK measure can be used instead.


$$APHR=2\times \frac{AP\times HLFRK}{AP+HLFRK}$$ (7)

#### 2.6.2. Twitter users’ evaluation

This approach mirrors the participants’ evaluation but focuses on Twitter users in the contemplation stage, identified using keywords related to Anorexia Nervosa. From 773 detected profiles, we selected 20 with the highest certainty (>0.95 probability) of being in the contemplation stage, ensuring a precise and relevant user sample for assessment. We then manually verified these users belonged to this group.

The rationale for limiting to 20 users is due to the extensive network analysis required. For each, we explored 200 followees to identify harmless ones, eventually expanding to a vast pool of 160,000 potential connections. For practicality and thorough evaluation, we narrowed this down to 100 random candidates per user, manageable for manual labeling and analysis.

The evaluation involved extracting users’ interest topics, identifying harmless candidates, and calculating similarities. We ranked these candidates, recommending the top 5, 10, 15, and 20 to each user, with 3 annotators determining the likelihood of follow-through, as the users could not personally participate in the evaluation. This method, chosen over direct followee testing, accounts for the low probability of users already following pro-recovery accounts, using existing harmless connections to define candidate pools. We use the same evaluation measures as described in Section 2.6.1.

*a) Twitter users’ evaluation baselines*. We compare the results of our approach with the baseline models described in Table 3. Notice that we consider Twitter’s recommender system as another baseline, but only to evaluate the types of users that are recommended by the platform, as it is our hypothesis that its recommendation approach puts on top of the suggestions for AN users accounts that are harmful for them.

**Table 3:** Baselines defined to evaluate the users’ recommendation approach.

Baseline model	Source of pool candidates	Types of users considered for the pool	Rank score per candidate of a given user
Model U.1	Sample of users obtained from the pool of candidates without considering the filtering step of our method.	Beneficial (0%), harmful (82%) and neutral (18%) users.	*Content*_*score*(u,*c*_*x*_)
Model U.2	Sample of users obtained from the pool of candidates of the user’s evaluation approach considering the filtering step of our method.	Beneficial (0%), harmful (30%) and neutral (70%) users.	*Content*_*score*(u,*c*_*x*_)
Model U.3	Sample of users obtained from the pool of candidates of the user’s evaluation approach considering the filtering step of our method.	Beneficial (0%), harmful (30%) and neutral (70%) users.	*harmlessness*_*score*(*c*_*x*_)×*Content*_*score*(u,*c*_*x*_)
Model U.4	Sample of twitter accounts that were labeled as either harmful, neutral or beneficial.	Equal number of beneficial, harmful, and neutral users	Random suggestions
Model U.5	Twitter’s recommender system’s pool of candidates.	Unknown	Unknown

Regarding model U.5, we analyzed Twitter’s recommendation method by evaluating the recommendations given by the platform (RQ4). The steps followed were: 1) among 50 twitter AN-Contemplation labeled accounts, we have labelled the followees (50 per each account) of the accounts as either harmful, neutral, or pro-recovery accounts. 2) We obtained the average number of accounts of each type followed. Then, 3) we have also created 20 Twitter accounts to reproduce the process of following accounts by ED users and evaluated the types of accounts suggested by Twitter to follow.

For each of the 20 accounts, we followed 50 accounts, from them, a percentage corresponded to harmless accounts and another percentage corresponded to harmful accounts (based on the ratios obtained from step 2). For the harmless accounts, users were followed based in the initial suggestions given by Twitter once an account is created. Regarding the harmful accounts, with the keywords: *edtwt*, *proana*, *promia* as search terms, we searched for harmful user’s accounts and followed randomly the corresponding percentage of accounts suggested according to the search terms. Later, based on these 50 accounts followed, we labeled the top 50 accounts suggested by Twitter in their “who to follow” section as either harmful, pro-recovery or neutral.

Notice that for the evaluation of this model we only compare with our model the percentages of harmful, beneficial, and neutral users suggested at *K* = 50. The choice of evaluating 50 followees for each of the 50 AN-Contemplation accounts was dictated by the need for a comprehensive yet feasible manual labeling effort, ensuring a representative sample of potential followee types. Similarly, the creation of 20 new Twitter accounts for simulating ED user behavior was constrained by Twitter’s account policies and the practicalities of manual analysis, balancing thoroughness with the logistical demands of the study.

### 2.7. Analysis of topics of interest

With the data collected from the survey applied to the participants with AN, we analyzed the results regarding their scores for each topic of interest (RQ1) (Table 1) and obtained the topics that are relevant for them by aggregating the results obtained by each participant and summarizing our findings in a boxplot. Regarding the topics of interest mostly related to AN for the participants, we analyzed the frequencies of terms used in the answers of users and represented these terms and their importance in a word cloud, where the terms or bigrams most used are displayed in major size. Finally, we established a comparative analysis of the topics of participants and Twitter users. We show the top 10 topics of interest for each group. Topics were ranked based in their frequencies. Following the same approach, we also obtained the top 10 terms most used by each group.

## 3. Results

### 3.1. Contemplation users’ detection

The results for the prediction of the Contemplation class over the test set are described in Table 4 (RQ2). The model selected as the best according to all measures, is the BoW model with a LR classifier. This was therefore the model used for the detection of Contemplation users in the recommender evaluation. The performance of the BoW model suggests that the vocabulary used by contemplation users is quite distinguishable from the one of control users. Notice the test set had 17 instances for the contemplation class, and 150 instances for the control class, which is why we focus on the results obtained for the contemplation class, instead of just considering the accuracy or the results for the control class. This same test set is used to evaluate the performance of all the models evaluated.

**Table 4:** Evaluation of contemplation users’ detection model.

Model	Classifier	Precision	Recall	F1-Score	Accuracy
Bag of words model	Logistic Regression	0.94	*0*.*94*	*0*.*94*	*0*.*98*
Lexicon model	Random Forest	0.92	0.71	0.80	0.96
CNN model	CNN	*1*	0.40	0.57	0.94

### 3.2. Harmless users’ detection

Our findings regarding the harmlessness classification model are described in Table 5 (RQ3), here we show the scores obtained for the ‘harmless’ class. The lexicon model obtained the best results for all the evaluation measures, and thus became the model used for the recommendation approach. The weakness of the BoW model may be given by the fact that in the dataset there are harmless and harmful users that make use of AN vocabulary. Therefore, it is likely for the Lexicon model to have identified more attributes that characterize harmless from harmful accounts. For this case, the test set had 55 instances of harmless cases and 76 instances of harmful cases.

**Table 5:** Harmless users’ detection models.

Model	Classifier	Precision	Recall	F1-Score	Accuracy
Bag of words model	SVM	0.69	0.85	0.76	0.78
Lexicon model	Random Forest	*0*.*79*	*0*.*95*	*0*.*86*	*0*.*87*
CNN model	CNN	0.68	0.74	0.71	0.74

#### 3.2.1. Survey participants’ evaluation

Results are described in Table 6. We can observe the results for the baseline models defined, and our proposal. We show results regarding Precision (P), Recall (R), Mean Average Precision (MAP), and pro-recovery suggested ratio (PRSR), neutral suggested ratio (NSR), harmful suggested ratio (HSR) and harmless suggested ratio (HLSR) of accounts at *K*. We also report the ratio of followed pro-recovery (PRFRS), neutral (NFRS), harmful (HFRS) and harmless (HLFRS) accounts over the number of accounts suggested of each type at K. Finally, we calculate the ratio of followed pro-recovery (PRFRK), harmless (HLFRK) and harmful (HFRK) accounts over the total number of accounts suggested (k), as described in Section 2.6.1. We also calculate the Average Precision-Harmlessness Ratio Score (APHR).

Regarding Precision, the baseline model V.5 performs better for every value of K and has the best MAP scores. However, this model does not consider any pro-recovery candidates. Regarding our approach, we can observe that there is a small difference in precision when compared with a model that only recommends harmful content (7% at worst, when K = 5). However, our proposal outperforms model V.4, which is the most similar to a common recommendation approach. We achieve an improvement in precision of up to 3% and, moreover, our method does not suggest any harmful accounts. Regarding recall (R), we can see that Models V.1, V.5 and our proposed approach obtain the best results depending on the value of K. Notice that, when K = 15, R is likely to be 1 as participants only annotated up to 15 suggestions per model. When it is not 1 is because R = 0 when no relevant suggestions have been made.

**Table 6:** Results obtained for the evaluation of the baselines and the proposed model for survey participants. We show results regarding Precision (P), Recall (R), Mean Average Precision (MAP), and pro-recovery suggested ratio (PRSR), neutral suggested ratio (NSR), harmful suggested ratio (HSR) and harmless suggested ratio (HLSR) of accounts at K accounts suggested. We also report the ratio of followed pro-recovery (PRFRS), neutral (NFRS), harmful (HFRS) and harmless (HLFRS) accounts over the number of accounts suggested of each type at K. We also calculate the ratio of followed pro-recovery (PRFRK), harmless (HLFRK) and harmful (HFRK) accounts over the total number of accounts suggested (k), along with the Average Precision-Harmlessness Ratio Score (APHR). In cursive, we highlight the best results for each combination of model and K.

Model	Description	K	P	R	MAP	PRSR	NSR	HSR	HLSR	PRFRS	NFRS	HFRS	HLFRS	PRFRK	HLFRK	HFRK	APHR
Model V.1	Only harmful accounts + content	5	0.25	0.36	0.16	0.00	0.00	*1*.*00*	0.00	0.00	0.00	0.25	0.00	0.00	0.00	*0*.*25*	0.00
		10	0.25	*0*.*78*	0.27	0.00	0.00	*1*.*00*	0.00	0.00	0.00	*0*.*25*	0.00	0.00	0.00	0.25	0.00
		15	0.24	*1*.*00*	0.35	0.00	0.00	*1*.*00*	0.00	0.00	0.00	*0*.*24*	0.00	0.00	0.00	*0*.*24*	0.00
Model V.2	Only beneficial accounts + content	5	0.25	0.30	0.16	*1*.*00*	0.00	0.00	*1*.*00*	*0*.*25*	0.00	0.00	0.25	*0*.*25*	0.25	0.00	0.20
		10	0.25	0.66	0.29	*1*.*00*	0.00	0.00	*1*.*00*	*0*.*25*	0.00	0.00	0.25	*0*.*25*	*0*.*25*	0.00	0.27
		15	0.18	0.75	0.31	*1*.*00*	0.00	0.00	*1*.*00*	0.18	0.00	0.00	0.18	*0*.*18*	0.18	0.00	0.23
Model V.3	equal harmful, neutral and beneficial + random	5	0.08	0.22	0.14	0.38	0.47	0.15	0.85	0.04	0.08	0.00	0.09	0.03	0.08	0.00	0.10
		10	0.11	0.64	0.20	0.33	0.46	0.21	0.79	0.17	0.11	0.06	0.13	0.05	0.10	0.01	0.13
		15	0.13	*1*.*00*	0.26	*0*.*35*	0.40	0.25	0.75	0.14	0.10	0.20	0.12	0.05	0.09	0.03	0.13
Model V.4	no filtering step + content score	5	0.18	0.34	0.14	0.00	0.30	*0*.*70*	0.30	0.00	0.08	0.16	0.08	0.00	0.05	0.13	0.07
		10	0.18	0.58	0.21	0.00	0.24	*0*.*76*	0.24	0.00	0.08	0.19	0.08	0.00	0.03	*0*.*15*	0.05
		15	0.17	0.88	0.27	0.00	0.25	*0*.*75*	0.25	0.00	0.17	0.15	0.17	0.00	0.04	0.13	0.07
Model V.5	filtering step + content score	5	*0*.*38*	*0*.*39*	*0*.*29*	0.00	*0*.*80*	0.20	0.80	0.00	*0*.*26*	*0*.*33*	*0*.*26*	0.00	*0*.*23*	0.15	*0*.*26*
		10	*0*.*33*	0.67	*0*.*43*	0.00	*0*.*73*	0.28	0.73	0.00	*0*.*26*	*0*.*18*	*0*.*26*	0.00	0.21	0.11	*0*.*28*
		15	*0*.*27*	0.88	*0*.*48*	0.00	0.68	0.33	0.68	0.00	*0*.*22*	*0*.*19*	*0*.*22*	0.00	0.17	0.10	0.25
Model proposed	Filtering step + content score + harmlessness factor + beneficial accounts	5	0.18	*0*.*39*	0.23	*0*.*73*	0.28	0.00	*1*.*00*	*0*.*20*	0.06	0.00	0.18	0.15	0.18	0.00	0.20
		10	0.21	0.71	0.32	*0*.*36*	0.64	0.00	*1*.*00*	*0*.*20*	0.21	0.00	0.21	0.08	0.21	0.00	0.25
		15	0.20	*1*.*00*	0.38	0.24	*0*.*75*	0.00	*0*.*99*	*0*.*20*	0.20	0.00	0.20	0.05	*0*.*20*	0.00	*0*.*26*

Our model notably suggests pro-recovery accounts primarily within the top 5 recommendations, with about 20% being followed. Specifically, in a pro-recovery-only scenario (Model V.2), 25% of suggestions at K = 10 and 18% at K = 15 are followed, indicating a high willingness among AN users to engage with such content, closely matching the interest in harmful accounts. In contrast, a typical recommender (Model V.4) shows a high harmful account suggestion rate (75% at K = 15) but doesn’t outperform our model in precision, recall, or MAP. The APHR metric further highlights our model’s effectiveness alongside model V.5, while Model V.1, not recommending any harmless accounts, fares the worst.

#### 3.2.2. Twitter users’ evaluation

Table 7 shows Twitter user evaluation results. Model U.1 (common recommender) scores highest in precision and recall but suggests the most harmful accounts. Model U.2, applying our filtering step, suggests the most neutral users. Models U.4 (at K = 15, K = 20) and our proposal (at K = 5, K = 10) lead in pro-recovery suggestions, with ours seeing higher follow rates due to non-random recommendations. Our model consistently results in the most harmless and pro-recovery follows across all Ks, showing an advantage over Model U.3 due to network features. The APHR score highlights our model’s balanced performance, and the precision gap with Model U.1 (17% at K = 20) is deemed reasonable given the safer account recommendations.

**Table 7:** Types of users followed by Twitter’s AN contemplation users.

Label	followees (mean)	followees (median)	followees (%)	users suggested (mean)	users suggested (median)	users suggested (%)
Harmful	40.74	41.5	81.48% (40.74/50)	36.85	36.5	73.70% (36.85/50)
Neutral	9.26	8.5	18.52% (9.26/50)	12.65	12.5	25.30% (12.65/50)
Pro-recovery	0	0	0% (0/50)	0.5	0	1% (0.5/50)

#### 3.2.3. Twitter’s recommender evaluation

Table 8 (RQ4) reveals that contemplation users on Twitter follow few harmless accounts (18.52%), with no pro-recovery accounts followed. On average, 73.70% of Twitter’s suggestions to AN individuals are likely harmful. Comparing Twitter’s model to ours at K = 50, our model suggests 21% beneficial, 47% neutral, and 32% harmful accounts on average.

**Table 8:** Results obtained for the evaluation of the baselines and the proposed model for users. We show results regarding Precision (P), Recall (R), Mean Average Precision (MAP), and pro-recovery suggested ratio (PRSR), neutral suggested ratio (NSR), harmful suggested ratio (HSR) and harmless suggested ratio (HLSR) of accounts at K accounts suggested. We also report the ratio of followed pro-recovery (PRFRS), neutral (NFRS), harmful (HFRS) and harmless (HLFRS) accounts over the number of accounts suggested of each type at K. We also calculate the ratio of followed pro-recovery (PRFRK), harmless (HLFRK) and harmful (HFRK) accounts over the total number of accounts suggested (K), along with the Average Precision-Harmlessness Ratio Score (APHR). In cursive, we highlight the best results for each combination of model and K.

Model	Description	K	P	R	MAP	PRSR	NSR	HSR	HLSR	PRFRS	NFRS	HFRS	HLFRS	PRFRK	HLFRK	HFRK	APHR
Model U.1	No filtering step + content score	5	*0*.*60*	*0*.*26*	0.21	0.00	0.08	*0*.*92*	0.08	0.00	0.13	*0*.*62*	0.13	0.00	0.03	*0*.*57*	0.05
		10	*0*.*61*	*0*.*52*	0.37	0.00	0.11	*0*.*90*	0.11	0.00	0.21	*0*.*65*	0.21	0.00	0.03	*0*.*58*	0.06
		15	*0*.*60*	*0*.*77*	0.54	0.00	0.12	*0*.*88*	0.12	0.00	0.15	*0*.*60*	0.15	0.00	0.02	*0*.*58*	0.04
		20	*0*.*58*	*1*.*00*	0.67	0.00	0.14	*0*.*86*	0.14	0.00	0.19	*0*.*63*	0.19	0.00	0.03	*0*.*55*	0.06
Model U.2	Filtering step + content score	5	0.50	0.27	0.19	0.07	*0*.*46*	0.47	0.53	0.03	*0*.*20*	0.44	0.21	0.01	0.18	0.32	0.18
		10	0.46	0.51	0.32	0.05	*0*.*49*	0.47	0.54	0.03	0.20	0.40	0.20	0.01	0.17	0.29	0.22
		15	0.46	0.75	0.44	0.04	*0*.*50*	0.46	0.54	0.03	0.21	0.42	0.21	0.00	0.16	0.30	0.23
		20	0.46	*1*.*00*	0.56	0.04	*0*.*51*	0.46	0.54	0.03	0.20	0.40	0.20	0.01	0.16	0.30	0.25
Model U.3	Filtering step + content score + harmlessness factor + beneficial accounts	5	0.34	0.25	0.16	0.76	0.17	0.07	0.93	0.31	0.11	0.20	0.32	0.25	0.30	0.04	0.21
		10	0.34	0.47	0.25	*0*.*45*	0.33	0.23	*0*.*78*	0.32	*0*.*23*	0.33	0.28	0.14	0.22	0.12	0.23
		15	0.36	0.74	0.35	0.30	0.43	0.27	0.73	0.32	0.28	0.34	0.31	0.09	0.22	0.14	0.27
		20	0.37	*1*.*00*	0.44	0.23	0.46	0.31	0.7	0.32	*0*.*28*	0.33	0.30	0.07	0.20	0.16	0.28
Model U.4	random recommendations	5	0.27	0.24	0.16	0.34	0.40	0.26	0.74	0.25	0.08	0.44	0.12	0.08	0.10	0.17	0.12
		10	0.26	0.49	0.24	0.33	0.42	0.26	0.75	0.24	0.09	0.53	0.14	0.07	0.11	0.15	0.15
		15	0.25	0.74	0.31	*0*.*31*	0.44	0.25	*0*.*75*	0.28	0.07	0.66	0.14	0.08	0.11	0.15	0.16
		20	0.25	0.95	0.37	*0*.*30*	0.45	0.25	*0*.*75*	0.29	0.07	0.61	0.14	0.08	0.11	0.14	0.17
Model proposed	Filtering step + content score + harmlessness factor + social network features + beneficial accounts	5	0.4	*0*.*26*	0.17	*0*.*81*	0.14	0.05	*0*.*95*	*0*.*44*	0.08	0.10	*0*.*40*	*0*.*36*	*0*.*38*	0.02	*0*.*23*
		10	0.36	0.46	0.26	*0*.*45*	0.34	0.22	*0*.*78*	*0*.*42*	0.19	0.27	*0*.*34*	*0*.*18*	*0*.*27*	0.10	*0*.*26*
		15	0.39	0.74	0.37	0.30	0.42	0.28	0.72	*0*.*42*	*0*.*31*	0.28	*0*.*37*	*0*.*12*	*0*.*26*	0.13	*0*.*31*
		20	0.41	*1*.*00*	0.48	0.23	0.46	0.31	0.69	*0*.*42*	*0*.*28*	0.33	*0*.*35*	*0*.*09*	*0*.*24*	0.17	*0*.*32*

### 3.3. Analysis of topics of interest

We compare the topics of interest of Twitter’s contemplation users, and those of participants (RQ1). These topics correspond to those extracted automatically. As it can be seen in Table 9, the top 10 topics of interest of users and survey participants are quite similar and can easily be related to Anorexia Nervosa. Moreover, four topics can be found in both groups. Regarding the terms mostly used by participants and users we can see again that most of them are related to Anorexia Nervosa.

**Table 9:** Most addressed topics and most terms used by contemplation Twitter users and survey participants.

Rank	Participants’ topics	Twitter users’ topics	Participants’ terms	Twitter users’ terms
1	*Nutrition*	*Software*	eating disorders (tca)	fasting (ayuno)
2	*Social Networks*	*Social Networks*	diets (dieta)	fat (gorda)
3	Medical terms	Twitter	Instagram	*calories (calorías)*
4	Culture sociology	*Food*	weight (peso)	I ate (comí)
5	*Software*	Vegetarian food	*calories (calorías)*	day (día)
6	*Food*	*Nutrition*	lose weight (adelgazar)	eating (comiendo)
7	Images storage	Diary food	food (alimentos)	pretty (lindo)
8	Diets	Fruits	exercise (ejercicios)	horas (hours)
9	Energy units	Culinary ingredients	series	say (decir)
10	measurement units	Internet	self-image (imagen)	horrible

**Fig 4:**
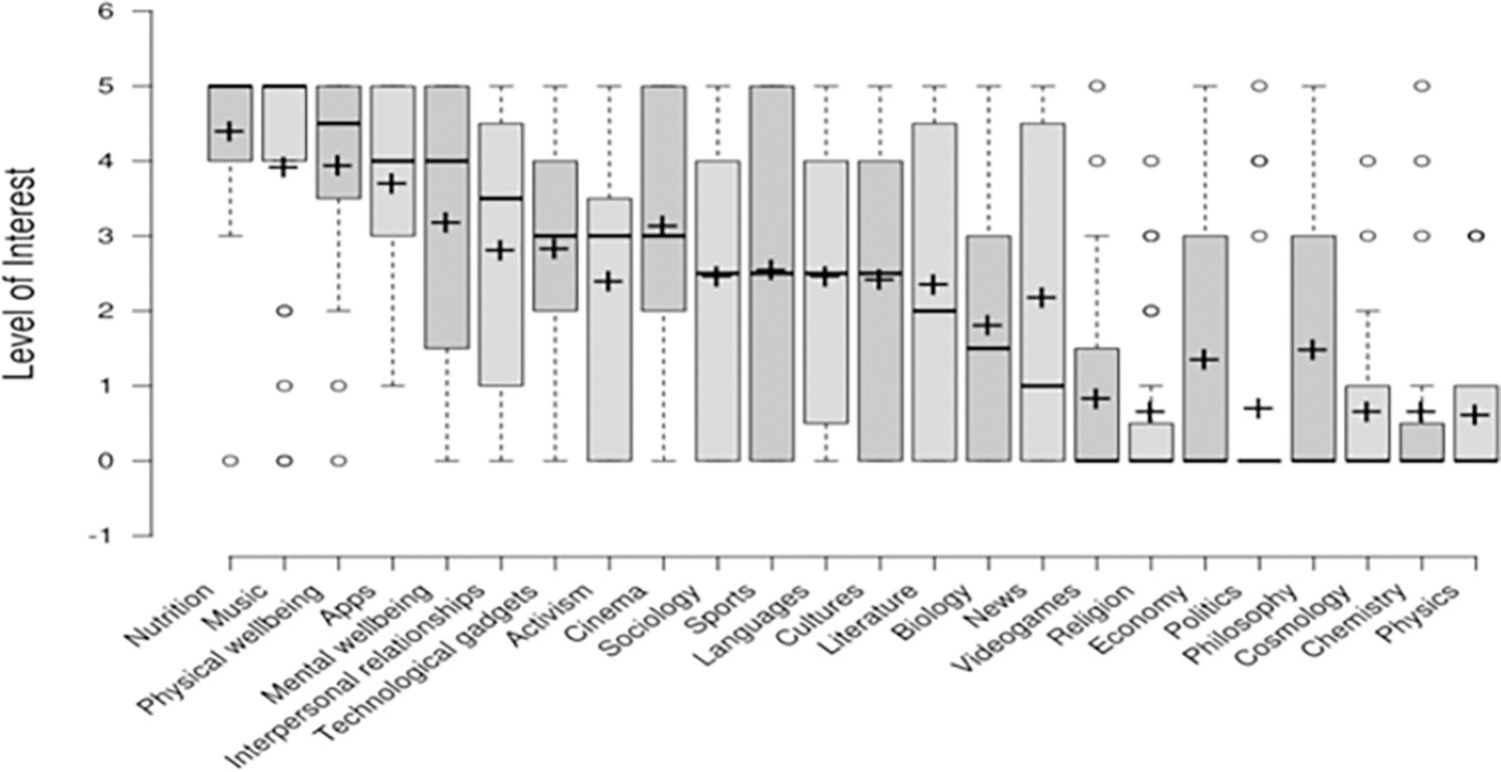
Levels of interest of participants in the predefined topics addressed in the survey.

About the main topics of interest of participants during the contemplation stage, in Fig 4 we report on the level of importance assigned to each subcategory predefined in Table 1. We observe in the boxplot that the main topics of interest are nutrition, music, physical wellbeing, apps, mental wellbeing, and interpersonal relationships. In addition, 77.27% (17/22) of the participants surveyed thought that the content suggested by social platforms was harmful for them.

**Fig 5:**
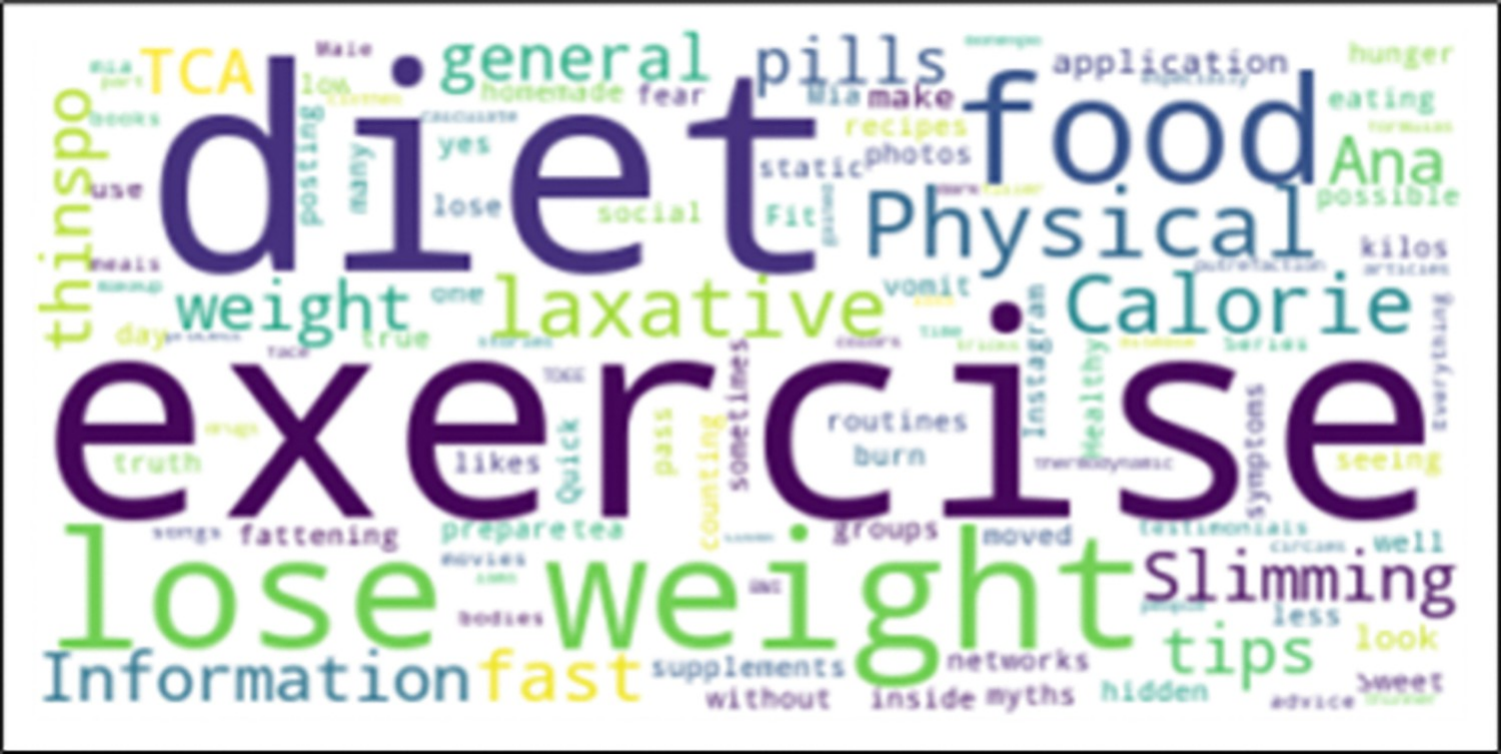
Word cloud obtained from the descriptions provided by survey participants (N = 22) of their topics of interest related to AN at the contemplation stage.

Also, the topics related to AN that they were most interested at are described in Fig 5, where we can see the top terms (translated from Spanish to English) used to describe their topics of interest. We can see in the word cloud that terms as exercise (freq = 9), diet (freq = 9), lose weight (freq = 9), food (freq = 6) and physical (freq = 4) are the most mentioned.

## 4. Discussion

### 4.1. Conclusions

Addressing our research questions, for RQ1, we discovered that the key interests of individuals with Anorexia Nervosa center around their condition, with a notable focus on nutrition and wellbeing. Music emerged as a significant non-condition-related interest, providing a potential pathway to connect users with harmless content.

In response to RQ2, we successfully identified users at the contemplation stage using a bag of words model, achieving a 0.94 F1 score, indicating its efficacy for this purpose.

For RQ3, we developed a classifier to distinguish harmless from harmful accounts with 87% accuracy, enhancing our recommendation system’s filtering process for efficiency, despite most harmful users following similar accounts.

Investigating RQ4 revealed that 81.48% of accounts followed by AN users are harmful, with Twitter’s recommendations also predominantly harmful, underscoring the need for safer alternatives.

Addressing RQ5, our recommender system significantly reduces harmful suggestions, offering 68% harmless content at minimum, outperforming Twitter and common models in fostering a safer online environment for AN users.

Regarding RQ6, results affirm that contemplation-stage users are inclined to follow harmless, including pro-recovery, accounts, validating the practicality of implementing such recommender systems on social platforms.

Finally, for RQ7, we introduced the APHR measure to assess recommender systems’ effectiveness in suggesting non-harmful content, prioritizing user safety alongside engagement metrics.

### 4.2. Limitations

A key challenge in our study was accessing individuals in the contemplation phase, as they are typically undiagnosed and not yet engaged with treatment organizations. Consequently, our participants were mainly in advanced treatment stages, possibly influencing their survey responses. However, their past contemplation phase experiences were valuable for our findings. Similarly, for user evaluations, we relied on annotators to infer user preferences. Additional limitations include the necessity of translating Spanish data for analysis and tool compatibility, the focus on Twitter which may not fully represent behaviors on other platforms, and potential biases from annotators and participants’ personal perspectives.

Our study’s scope was also constrained by the sample size, particularly due to the manual labeling required for much of our data analysis, which is labor-intensive and time-consuming. Additionally, the reliance on volunteer participation for our surveys introduced further limitations, as our ability to gather data was directly dependent on the willingness and availability of individuals to engage with our study.

### 4.3. Future work

Our findings are relevant for the design of recommender systems that are aware of the issues of excessive personalization for people with mental disorders. Future work shall address related mental health issues, such as depression or suicidal ideation, to analyze the impact of such systems in different types of users.

Having demonstrated the viability of our approach in encouraging the target users to accept recommendations, we acknowledge the framework’s potential. With this results, future iterations of this research shall delve into a broader array of predictive models and machine learning algorithms, including but not limited to advanced deep learning frameworks like transformers. This expansion aims to refine and enhance the accuracy and efficacy of our recommendation approach. Furthermore, we plan to conduct comprehensive ablation studies to critically assess the impact of various components within our models, particularly in relation to the ranking index. Such analyses will provide deeper insights into the essential elements of our approach, guiding targeted improvements and ensuring a more robust and effective system.
